# Supplementary material for: Vaccinia Virus Attenuation by Codon Deoptimization of the A24R Gene for Vaccine Development
Source: Microbiol Spectr. 2022 May 18;10(3):e00272-22. doi: 10.1128/spectrum.00272-22 (PMC9241885; doi:10.1128/spectrum.00272-22)
Supplement: SUPPLEMENTAL FILE 1 — Supplemental material. Download spectrum.00272-22-s001.pdf, PDF file, 0.1 MB [file spectrum.00272-22-s001.pdf]

Supplementary Figure 1. Nucleotide sequence of wild type (top) and CD (bottom) VV A24R.
Red indicates nucleotide changes introduced to CD VV A24R.

```
A24R   ATG AAA AAA AAC ACT GAT TCA GAA ATG GAT CAA CGA CTA GGG TAT AAG TTT
CD     ATG AAA AAA AAT ACG GAT TCG GAA ATG GAT CAA CGT CTA GGT TAT AAA TTT

A24R   TTG GTG CCT GAT CCT AAA GCC GGA GTT TTT TAT AGA CCG TTA CAT TTC CAA
CD     CTA GTA CCG GAC CCG AAA GCG GGT GTA TTT TAT CGT CCG CTA CAT TTT CAA

A24R   TAT GTA TCG TAT TCT AAT TTT ATA TTG CAT CGA TTG CAT GAA ATC TTG ACC
CD     TAT GTA TCG TAT TCG AAT TTT ATA CTA CAT CGT CTA CAT GAA ATA CTA ACG

A24R   GTC AAG CGG CCA CTC TTA TCG TTT AAG AAT AAT ACA GAA CGA ATT ATG ATA
CD     GTA AAA CGT CCG CTA CTA TCG TTT AAA AAT AAT ACG GAA CGT ATA ATG ATA

A24R   GAA ATT AGC AAT GTT AAA GTG ACT CCT CCA GAT TAC TCA CCT ATA ATC GCG
CD     GAA ATA TCG AAT GTA AAA GTA ACG CCG CCG GAT TAT TCG CCG ATA ATA GCG

A24R   AGT ATT AAA GGT AAG AGT TAT GAT GCA TTA GCC ACG TTC ACT GTA AAT ATC
CD     TCG ATA AAA GGT AAA TCG TAT GAT GCG CTA GCG ACG TTT ACG GTA AAT ATA

A24R   TTT AAA GAG GTA ATG ACC AAA GAG GGT ATA TCC ATC ACT AAA ATA AGT AGT
CD     TTT AAA GAA GTA ATG ACG AAA GAA GGT ATA TCG ATA ACG AAA ATA TCG TCG

A24R   TAT GAG GGA AAA GAT TCT CAT TTG ATA AAA ATT CCG CTA CTA ATA GGA TAC
CD     TAT GAA GGT AAA GAT TCG CAT CTA ATA AAA ATA CCG CTA CTA ATA GGT TAT

A24R   GGG AAT AAA AAT CCA CTT GAT ACA GCC AAG TAT CTT GTT CCT AAT GTC ATA
CD     GGT AAT AAA AAT CCG CTA GAT ACG GCG AAA TAT CTA GTA CCG AAT GTA ATA

A24R   GGT GGA GTC TTT ATC AAT AAA CAA TCT GTC GAA AAA GTA GGA ATT AAT CTA
CD     GGT GGT GTA TTT ATA AAT AAA CAA TCG GTA GAA AAA GTA GGT ATA AAT CTA

A24R   GTA GAA AAG ATT ACA ACA TGG CCA AAA TTT AGG GTT GTT AAG CCA AAC TCA
CD     GTA GAA AAA ATA ACG ACG TGG CCG AAA TTT CGT GTA GTA AAA CCG AAT TCG

A24R   TTC ACT TTC TCG TTT TCC TCC GTA TCC CCT CCT AAT GTA TTA CCG ACA AGA
CD     TTT ACG TTT TCG TTT TCG TCG GTA TCG CCG CCG AAT GTA CTA CCG ACG CGT

A24R   TAT CGC CAT TAC AAG ATA TCT CTG GAT ATA TCA CAA TTG GAA GCG TTG AAT
CD     TAT CGT CAT TAT AAA ATA TCG CTA GAT ATA TCG CAA CTA GAA GCG CTA AAT

A24R   ATA TCA TCG ACA AAG ACA TTT ATA ACG GTC AAT ATT GTT TTG CTG TCT CAA
CD     ATA TCG TCG ACG AAA ACG TTT ATA ACG GTA AAT ATA GTA CTA CTA TCG CAA

A24R   TAT TTA TCT AGA GTG AGT CTA GAA TTC ATT AGA CGT AGT TTA TCA TAC GAT
CD     TAT CTA TCG CGT GTA TCG CTA GAA TTT ATA CGT CGT TCG CTA TCG TAT GAT

A24R   ATG CCT CCA GAA GTT GTC TAT CTA GTA AAC GCG ATA ATA GAT AGT GCT AAA
CD     ATG CCG CCG GAA GTA GTA TAT CTA GTA AAT GCG ATA ATA GAT TCG GCG AAA
```

```
A24R   CGA ATT ACT GAA TCT ATT ACT GAC TTT AAT ATT GAT ACA TAC ATT AAT GAC
CD     CGT ATA ACG GAA TCG ATA ACG GAT TTT AAT ATA GAT ACG TAT ATA AAT GAT

A24R   CTG GTG GAA GCT GAA CAC ATT AAA CAA AAA TCT CAG TTA ACG ATC AAC GAG
CD     CTA GTA GAA GCG GAA CAT ATA AAA CAA AAA TCG CAA CTA ACG ATA AAT GAA

A24R   TTC AAA TAT GAA ATG CTG CAT AAC TTT TTA CCT CAT ATG AAC TAT ACA CCC
CD     TTT AAA TAT GAA ATG CTA CAT AAT TTT CTA CCG CAT ATG AAT TAT ACG CCG

A24R   GAT CAA CTA AAG GGA TTT TAT ATG ATA TCT TTA CTA AGA AAG TTT CTC TAC
CD     GAT CAA CTA AAA GGT TTT TAT ATG ATA TCG CTA CTA CGT AAA TTT CTA TAT

A24R   TGT ATC TAC CAC ACT TCT AGA TAT CCA GAT AGA GAT TCG ATG GTT TGT CAT
CD     TGT ATA TAT CAT ACG TCG CGT TAT CCG GAT CGT GAT TCG ATG GTA TGT CAT

A24R   CGC ATC CTA ACG TAC GGC AAA TAT TTT GAG ACG TTG GCA CAT GAT GAA TTA
CD     CGT ATA CTA ACG TAT GGT AAA TAT TTT GAA ACG CTA GCG CAT GAT GAA CTA

A24R   GAG AAT TAC ATA GGC AAC ATC CGA AAC GAT ATC ATG AAC AAT CAC AAG AAC
CD     GAA AAT TAT ATA GGT AAT ATA CGT AAT GAT ATA ATG AAT AAT CAT AAA AAT

A24R   AGA GGC ACT TAC GCG GTA AAC ATT CAT GTA CTA ACA ACT CCC GGA CTT AAT
CD     CGT GGT ACG TAT GCG GTA AAT ATA CAT GTA CTA ACG ACG CCG GGT CTA AAT

A24R   CAC GCG TTT TCT AGC TTA TTG AGT GGA AAG TTC AAA AAG TCA GAC GGT AGT
CD     CAT GCG TTT TCG TCG CTA CTA TCG GGT AAA TTT AAA AAA TCG GAT GGT TCG

A24R   TAT CGA ACA CAT CCT CAC TAT TCA TGG ATG CAG AAT ATT TCT ATT CCT AGG
CD     TAT CGT ACG CAT CCG CAT TAT TCG TGG ATG CAA AAT ATA TCG ATA CCG CGT

A24R   AGT GTT GGA TTT TAT CCG GAT CAA GTA AAG ATT TCA AAG ATG TTT TCT GTC
CD     TCG GTA GGT TTT TAT CCG GAT CAA GTA AAA ATA TCG AAA ATG TTT TCG GTA

A24R   AGA AAA TAC CAT CCA AGT CAA TAT CTT TAC TTT TGT TCA TCG GAC GTT CCG
CD     CGT AAA TAT CAT CCG TCG CAA TAT CTA TAT TTT TGT TCG TCG GAT GTA CCG

A24R   GAA AGA GGT CCT CAG GTA GGT TTA GTA TCT CAA TTG TCT GTC TTG AGT TCC
CD     GAA CGT GGT CCG CAA GTA GGT CTA GTA TCG CAA CTA TCG GTA CTA TCG TCG

A24R   ATT ACA AAT ATA CTA ACG TCT GAG TAT TTG GAT TTG GAA AAG AAA ATT TGT
CD     ATA ACG AAT ATA CTA ACG TCG GAA TAT CTA GAT CTA GAA AAA AAA ATA TGT

A24R   GAG TAT ATC AGA TCA TAT TAT AAA GAT GAT ATA AGT TAC TTT GAA ACA GGA
CD     GAA TAT ATA CGT TCG TAT TAT AAA GAT GAT ATA TCG TAT TTT GAA ACG GGT

A24R   TTT CCA ATC ACT ATA GAA AAT GCT CTA GTC GCA TCT CTT AAT CCA AAT ATG
CD     TTT CCG ATA ACG ATA GAA AAT GCG CTA GTA GCG TCG CTA AAT CCG AAT ATG


A24R   ATA TGT GAT TTT GTA ACT GAC TTT AGA CGT AGA AAA CGG ATG GGA TTC TTC
CD     ATA TGT GAT TTT GTA ACG GAT TTT CGT CGT CGT AAA CGT ATG GGT TTT TTT
```

```
A24R   GGT AAC TTG GAG GTA GGT ATT ACT TTA GTT AGG GAT CAC ATG AAT GAA ATT
CD     GGT AAT CTA GAA GTA GGT ATA ACG CTA GTA CGT GAT CAT ATG AAT GAA ATA

A24R   CGC ATT AAT ATT GGA GCG GGA AGA TTA GTC AGA CCA TTC TTG GTT GTG GAT
CD     CGT ATA AAT ATA GGT GCG GGT CGT CTA GTA CGT CCG TTT CTA GTA GTA GAT

A24R   AAC GGA GAG CTC ATG ATG GAT GTG TGT CCG GAG TTA GAA AGC AGA TTA GAC
CD     AAT GGT GAA CTA ATG ATG GAT GTA TGT CCG GAA CTA GAA TCG CGT CTA GAT

A24R   GAC ATG ACA TTC TCT GAC ATT CAG AAA GAG TTT CCG CAT GTC ATC GAA ATG
CD     GAT ATG ACG TTT TCG GAT ATA CAA AAA GAA TTT CCG CAT GTA ATA GAA ATG

A24R   GTA GAT ATA GAA CAA TTT ACT TTT AGT AAC GTA TGT GAA TCG GTT CAA AAA
CD     GTA GAT ATA GAA CAA TTT ACG TTT TCG AAT GTA TGT GAA TCG GTA CAA AAA

A24R   TTT AGA ATG ATG TCA AAG GAT GAA AGA AAG CAA TAC GAT TTA TGT GAC TTT
CD     TTT CGT ATG ATG TCG AAA GAT GAA CGT AAA CAA TAT GAT CTA TGT GAT TTT

A24R   CCT GCC GAA TTT AGA GAT GGA TAT GTG GCA TCT TCA TTA GTG GGA ATC AAT
CD     CCG GCG GAA TTT CGT GAT GGT TAT GTA GCG TCG TCG CTA GTA GGT ATA AAT

A24R   CAC AAT TCT GGA CCC AGA GCT ATT CTT GGA TGT GCT CAA GCT AAA CAA GCT
CD     CAT AAT TCG GGT CCG CGT GCG ATA CTA GGT TGT GCG CAA GCG AAA CAA GCG

A24R   ATC TCT TGT CTG AGT TCG GAT ATA CGA AAT AAA ATA GAC AAT GGA ATT CAT
CD     ATA TCG TGT CTA TCG TCG GAT ATA CGT AAT AAA ATA GAT AAT GGT ATA CAT

A24R   TTG ATG TAT CCA GAG AGG CCA ATC GTG ATT AGT AAG GCT TTA GAA ACT TCA
CD     CTA ATG TAT CCG GAA CGT CCG ATA GTA ATA TCG AAA GCG CTA GAA ACG TCG

A24R   AAG ATT GCG GCT AAT TGC TTC GGC CAA CAT GTT ACT ATA GCA TTA ATG TCG
CD     AAA ATA GCG GCG AAT TGT TTT GGT CAA CAT GTA ACG ATA GCG CTA ATG TCG

A24R   TAC AAA GGT ATC AAT CAA GAG GAT GGA ATT ATC ATC AAA AAA CAA TTT ATT
CD     TAT AAA GGT ATA AAT CAA GAA GAT GGT ATA ATA ATA AAA AAA CAA TTT ATA

A24R   CAG AGA GGC GGT CTC GAT ATA GTT ACC GCA AAG AAA CAT CAA GTA GAA ATT
CD     CAA CGT GGT GGT CTA GAT ATA GTA ACG GCG AAA AAA CAT CAA GTA GAA ATA

A24R   CCG TTG GAA AAC TTT AAT AAC AAA GAA AGA GAT AGG TCT AAC GCC TAT TCA
CD     CCG CTA GAA AAT TTT AAT AAT AAA GAA CGT GAT CGT TCG AAT GCG TAT TCG

A24R   AAA TTA GAA AGT AAT GGA TTA GTT AGA CTG AAT GCT TTC TTG GAA TCC GGA
CD     AAA CTA GAA TCG AAT GGT CTA GTA CGT CTA AAT GCG TTT CTA GAA TCG GGT

A24R   GAC GCT ATG GCA CGA AAT ATC TCA TCA AGA ACT CTT GAA GAT GAT TTT GCT
CD     GAT GCG ATG GCG CGT AAT ATA TCG TCG CGT ACG CTA GAA GAT GAT TTT GCG


A24R   AGA GAT AAT CAG ATT AGC TTC GAT GTT TCC GAG AAA TAT ACC GAT ATG TAC
CD     CGT GAT AAT CAA ATA TCG TTT GAT GTA TCG GAA AAA TAT ACG GAT ATG TAT
```

**A24R**	AAA TCT CGC GTT GAA CGA GTA CAA GTA GAA CTT ACT GAC AAA GTT AAG GTA
**CD**	AAA TCG CGT GTA GAA CGT GTA CAA GTA GAA CTA ACG GAT AAA GTA AAA GTA
**A24R**	CGA GTA TTA ACC ATG AAA GAA AGA AGA CCC ATT CTA GGA GAT AAA TTT ACC
**CD**	CGT GTA CTA ACG ATG AAA GAA CGT CGT CCG ATA CTA GGT GAT AAA TTT ACG
**A24R**	ACT AGA ACG AGT CAA AAG GGA ACA GTC GCG TAT GTC GCG GAT GAA ACG GAA
**CD**	ACG CGT ACG TCG CAA AAA GGT ACG GTA GCG TAT GTA GCG GAT GAA ACG GAA
**A24R**	CTT CCA TAC GAC GAA AAT GGT ATC ACA CCA GAT GTC ATT ATT AAT TCT ACA
**CD**	CTA CCG TAT GAT GAA AAT GGT ATA ACG CCG GAT GTA ATA ATA AAT TCG ACG
**A24R**	TCC ATC TTC TCT AGA AAA ACT ATA TCT ATG TTG ATA GAG GTT ATT TTA ACA
**CD**	TCG ATA TTT TCG CGT AAA ACG ATA TCG ATG CTA ATA GAA GTA ATA CTA ACG
**A24R**	GCC GCA TAT TCT GCT AAG CCG TAC AAC AAT AAG GGA GAA AAC CGA CCT GTC
**CD**	GCG GCG TAT TCG GCG AAA CCG TAT AAT AAT AAA GGT GAA AAT CGT CCG GTA
**A24R**	TGT TTT CCT AGT AGT AAC GAA ACA TCC ATC GAT ACA TAT ATG CAA TTC GCT
**CD**	TGT TTT CCG TCG TCG AAT GAA ACG TCG ATA GAT ACG TAT ATG CAA TTT GCG
**A24R**	AAA CAA TGT TAT GAG CAT TCA AAT CCG AAA TTG TCC GAT GAA GAA TTA TCG
**CD**	AAA CAA TGT TAT GAA CAT TCG AAT CCG AAA CTA TCG GAT GAA GAA CTA TCG
**A24R**	GAT AAA ATC TTT TGT GAA AAG ATT CTC TAT GAT CCT GAA ACG GAT AAG CCT
**CD**	GAT AAA ATA TTT TGT GAA AAA ATA CTA TAT GAT CCG GAA ACG GAT AAA CCG
**A24R**	TAT GCA TCC AAA GTA TTT TTT GGA CCA ATT TAT TAC TTG CGT CTG AGG CAT
**CD**	TAT GCG TCG AAA GTA TTT TTT GGT CCG ATA TAT TAT CTA CGT CTA CGT CAT
**A24R**	TTA ACT CAG GAC AAG GCA ACC GTT AGA TGT AGA GGT AAA AAG ACG AAG CTC
**CD**	CTA ACG CAA GAT AAA GCG ACG GTA CGT TGT CGT GGT AAA AAA ACG AAA CTA
**A24R**	ATT AGA CAG GCG AAT GAG GGA CGA AAA CGT GGA GGA GGT ATC AAG TTC GGA
**CD**	ATA CGT CAA GCG AAT GAA GGT CGT AAA CGT GGT GGT GGT ATA AAA TTT GGT
**A24R**	GAA ATG GAG AGA GAC TGT TTA ATA GCG CAT GGC GCA GCC AAT ACT ATT ACA
**CD**	GAA ATG GAA CGT GAT TGT CTA ATA GCG CAT GGT GCG GCG AAT ACG ATA ACG
**A24R**	GAA GTT TTA AAA GAC TCA GAA GAG GAT TAT CAA GAT GTG TAT GTT TGT GAA
**CD**	GAA GTA CTA AAA GAT TCG GAA GAA GAT TAT CAA GAT GTA TAT GTA TGT GAA
**A24R**	AAT TGT GGA GAC ATA GCA GCA CAA ATC AAG GGT ATT AAT ACA TGT CTT AGA
**CD**	AAT TGT GGT GAT ATA GCG GCG CAA ATA AAA GGT ATA AAT ACG TGT CTA CGT
**A24R**	TGT TCA AAA CTT AAT CTC TCT CCT CTC TTA ACA AAA ATT GAT ACC ACG CAC
**CD**	TGT TCG AAA CTA AAT CTA TCG CCG CTA CTA ACG AAA ATA GAT ACG ACG CAT
**A24R**	GTA TCT AAA GTA TTT CTT ACT CAA ATG AAC GCC AGA GGC GTA AAA GTC AAA
**CD**	GTA TCG AAA GTA TTT CTA ACG CAA ATG AAT GCG CGT GGT GTA AAA GTA AAA

A24R TTA GAT TTC GAA CGA AGG CCT CCT TCG TTT TAT AAA CCA TTA GAT AAA GTT
CD CTA GAT TTT GAA CGT CGT CCG CCG TCG TTT TAT AAA CCG CTA GAT AAA GTA

A24R GAT CTC AAG CCG TCT TTT CTG GTG TAA
CD GAT CTA AAA CCG TCG TTT CTA GTA TAA
